# Autosomal dominant polycystic kidney disease with diffuse proliferative glomerulonephritis - an unusual association: a case report and review of the literature

**DOI:** 10.1186/1752-1947-4-125

**Published:** 2010-04-29

**Authors:** Sanjay D'Cruz, Rajdeep Singh, Harsh Mohan, Ravinder Kaur, Ranjana Walker Minz, Vinay Kapoor, Atul Sachdev

**Affiliations:** 1 Department of Medicine, Government Medical College & Hospital, Chandigarh 160030, India; 2 Department of Surgery, Government Medical College & Hospital, Chandigarh 160030, India; 3 Department of Pathology, Government Medical College & Hospital, Chandigarh 160030, India; 4 Department of Radiodiagnosis, Government Medical College & Hospital, Chandigarh 160030, India; 5 Department of Immunopathology, PGIMER, Chandigarh 160012, India

## Abstract

**Introduction:**

Autosomal dominant polycystic kidney disease is an inherited disorder that is characterized by the development and growth of cysts in the kidneys and other organs. Urinary protein excretion is usually less than 1 g/24 hours in autosomal dominant polycystic kidney disease, and an association of nephrotic syndrome with this condition is considered rare. There are only anecdotal case reports of autosomal dominant polycystic kidney disease associated with nephrotic syndrome, with focal segmental glomerulosclerosis being the most commonly reported histopathological diagnosis. Nephrotic-range proteinuria in the presence of autosomal dominant polycystic kidney disease, with or without an accompanying decline in renal function, should be investigated by open renal biopsy to exclude coexisting glomerular disease. To the best of our knowledge, this is the first case of autosomal dominant polycystic kidney disease with histologically proven diffuse proliferative glomerulonephritis presenting with nephrotic-range proteinuria. No other reports of this could be found in a global electronic search of the literature.

**Case presentation:**

We report the case of a 35-year-old Indo-Aryan man with autosomal dominant polycystic kidney disease associated with nephrotic syndrome and a concomitant decline in his glomerular filtration rate. Open renal biopsy revealed diffuse proliferative glomerulonephritis. An accurate diagnosis enabled us to manage him conservatively with a successful outcome, without the use of corticosteroid which is the standard treatment and the drug most commonly used to treat nephrotic syndrome empirically.

**Conclusion:**

Despite the reluctance of physicians to carry out a renal biopsy on patients with autosomal dominant polycystic kidney disease, our case supports the idea that renal biopsy is needed in patients with polycystic kidney disease with nephrotic-range proteinuria to make an accurate diagnosis. It also illustrates the importance of open renal biopsy in planning appropriate treatment for patients with autosomal dominant polycystic kidney disease with nephrotic-range proteinuria. The treatment for various histological subtypes leading to nephrotic syndrome is different, and in this modern era we should practice evidence-based medicine and should avoid empirical therapy with its associated adverse effects.

## Introduction

In patients with autosomal dominant polycystic kidney disease (ADPKD), urinary protein excretion is usually less than 1 g/24 hours. If proteinuria reaches the nephrotic range, the possibility of another coexistent glomerular disease should always be considered. In such a situation, open renal biopsy is warranted to reach a firm diagnosis. A review of the literature revealed only anecdotal case reports of ADPKD associated with nephrotic syndrome, with focal segmental glomerulosclerosis (FSGS) being the most commonly reported histopathological diagnosis. We report an unusual case of ADPKD with diffuse proliferative glomerulonephritis (DPGN) and nephrotic-range proteinuria. This is the first documented case of ADPKD with DPGN as no other reports of this could be found in a global electronic search of the literature.

## Case presentation

A 35-year-old Indo-Aryan man presented with facial puffiness and dyspnea on exertion for two weeks. He denied any history of abdominal pain, fever, dysuria, smoky urine, gross hematuria, sore throat, skin infection, joint pains, skin rash and oral ulcers. He was diagnosed with ADPKD, and his mother also had the disease. A general physical examination revealed his blood pressure was 170/110 mmHg and he had bilateral pitting pedal edema. A systemic examination of our patient was unremarkable except for bilaterally palpable knobby kidneys.

His blood test results showed the following: hemoglobin 136 g/L, total leukocyte count 8200/mm^3 ^(Neutrophils 65%, Lymphocytes 28%, Monocytes 5%, Eosinophils 2%), platelet count 2.5 × 10^5^/mm^3^, blood urea nitrogen 6.6 mmol/L, serum creatinine 150 μmol/L, serum potassium 3.8 mmol/L, serum albumin 30 g/L and serum cholesterol 7.42 mmol/L. Urine analysis showed 3+ albumin, and 20-25 leukocytes and 8-10 red blood cells per high power field. His 24-hour urinary protein excretion was 4.67 g. Urine culture and throat swab culture were sterile. Blood cultures on two separate occasions were also sterile. Ultrasound analysis of the abdomen of our patient showed an enlarged liver with multiple cysts and both kidneys were enlarged with multiple cysts of varying sizes (in both the cortex and medullary regions). Transthoracic echocardiography was normal and did not reveal any vegetations. Serology for hepatitis B surface antigen and anti-hepatitis C antibodies were non-reactive. Anti-nuclear antibody (ANA) and anti-neutrophil cytoplasmic antibody (ANCA) were shown as negative using the immunofluorescence technique. Anti-streptolysin O (ASO) titer was 350 Todd units. Serum complement (C3) was 70 mg/dL (normal range 75-135 mg/dL) which returned to normal levels at 12 weeks from the time of diagnosis.

**Figure 1:**
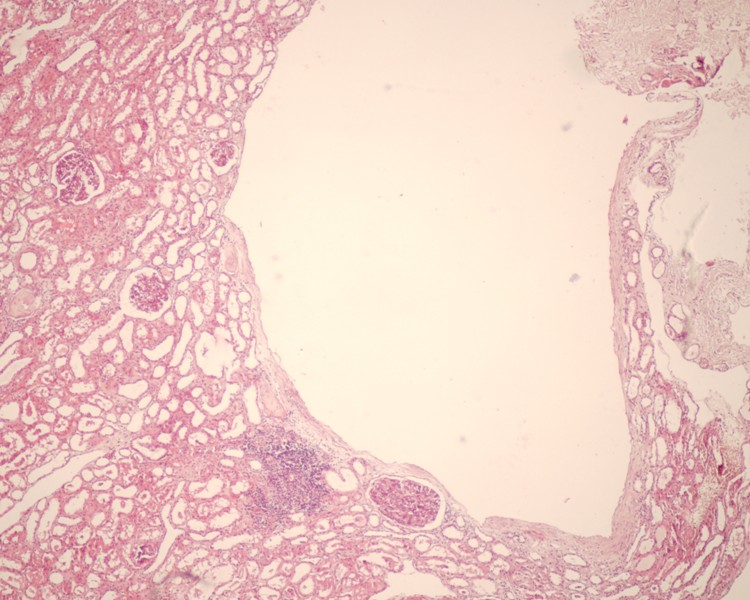
**Photomicrograph shows a cyst lined by flattened cells**. Renal parenchyma shows four glomeruli, a focus of lymphocytic aggregate in the interstitium and tubular atrophy. (hematoxylin and eosin, 100×)

**Figure 2:**
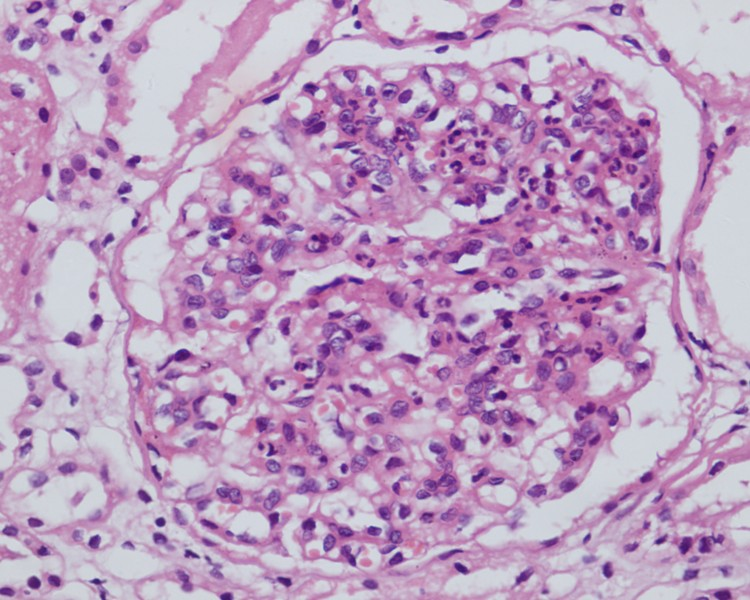
**The glomerulus shows mesangial hypercellularity, endocapillary proliferation and neutrophilic infiltrate (hematoxylin and eosin, 400×)**.

An open renal biopsy was carried out on our patient. Under low magnification light microscopy, cysts lined by flattened cells were observed. A total of 56 glomeruli were seen with variable mesangial hypercellularity and endocapillary proliferation with neutrophilic infiltration. There was focal tubular atrophy, and focal lymphocytic infiltrates were present in the interstitium. Blood vessels revealed intimal sclerosis (Figures 1 and 2). Immunofluorescence studies showed 20 glomeruli of which one was sclerosed. Focal immunoglobulin G (IgG) deposits (+ to ++), traces of immunoglobulin M (IgM) along with diffuse C3 (+++) deposits in a lumpy bumpy pattern were seen in the glomeruli. A diagnosis of ADPKD with post-streptococcal DPGN was made. Our patient was managed conservatively. This included close monitoring of his blood pressure, renal function tests and serum potassium along with salt restriction, diuretics and antihypertensive medications (losartan potassium and amlodipine). Corticosteroid was not prescribed for our patient. He improved over the next 12 weeks. His proteinuria returned to less than 1 g/24 hours with a normalization of renal function and a reduction in the requirement of antihypertensive medications.

## Discussion

The frequency of occurrence of proteinuria in ADPKD ranged from 14 to 34% in non-uremic adults to about 80% in adults with advanced renal failure in the review by Chapman *et al*. [1]. When present in patients with ADPKD, proteinuria is generally less than 1 g/24 hours. In patients of ADPKD; nephrotic range proteinuria, with or without an accompanying decline in renal function, is unusual and needs to be investigated further to exclude coexisting glomerular disease. Moreover, proteinuria hastens the progression of ADPKD to end-stage renal disease (ESRD) if it is untreated. Patients with established proteinuria have significantly higher mean arterial pressure, large renal volumes, lower creatinine clearances and a more aggressive course. Even microalbuminuric ADPKD patients with hypertension have a significantly higher filtration fraction and larger renal volumes [1].

The presence of nephrotic-range proteinuria is exceptional in ADPKD, with less than 20 cases having been reported in the literature so far. This may be due in part to the reluctance of nephrologists to perform renal biopsy in these patients, as this usually entails an open renal biopsy. Dalgaard *et al*. [2] described three cases of proteinuria >5 g/day in a report of 122 cases with ADPKD. Unfortunately renal biopsy data are not available in this series.

The various histopathological lesions reported in ADPKD patients are FSGS, membranous nephropathy, minimal change disease, crescentic glomerulonephritis, immunoglobulin A (IgA) nephropathy, mesangioproliferative glomerulonephritis, diabetic glomerulosclerosis and amyloidosis. Various histological subtypes that have been described in the literature are summarized in Table 1. A review of the literature revealed focal glomerulosclerosis to be the most common histological subtype associated with ADPKD, followed by membranous nephropathy and minimal change disease [3]. It is difficult to be certain whether these associations are coincidental or whether they demonstrate a specific pathogenetic relationship with ADPKD. The frequency of FSGS in the reported cases (5/18, 28%) is almost twice as high as the 15% frequency of FSGS found in the general adult population. By contrast, membranous nephropathy, the most common cause of idiopathic nephrotic syndrome in adults, with a frequency of 25%, was found in 16% (3/18) of the ADPKD patients with nephrotic syndrome, which suggests that FSGS may be more than a coincidental finding.

**Table 1:** Renal histology in cases of nephrotic-range proteinuria in autosomal dominant polycystic kidney disease patients reported in the literature so far.

**No**.	First author [Ref. No.] Year	Renal histopathology
1	Kida [6] 1972	Focal glomerulosclerosis and/or minimal change disease
2	Murphy [7] 1990	Focal glomerulosclerosis
3	Montoyo [4] 1992	Focal glomerulosclerosis
4	Dionisio [8] 1993	Focal glomerulosclerosis
5	Contreas [3] 1995	Focal glomerulosclerosis
6	Abe [9] 1985	Membranous glomerulonephritis
7	Shikata [10] 1991	Membranous glomerulonephritis
8	Saxena [11] 1993	Membranous glomerulonephritis
9	Nakahama [12] 1991	Minimal change disease
10	Kuroki [13] 1995	Minimal change disease
11	Panisello [14] 1988	Immunoglobulin A nephropathy
12	Hiura [15] 2006	Immunoglobulin A nephropathy
13	Licina [16] 1981	Crescentic glomerulonephritis
14	Hariharan [17] 1987	Intercapillary diabetic glomerulosclerosis
15	Villar [18] 1992	Type 1 membranoproliferative glomerulonephritis
16	Villar [18] 1992	Mesangioproliferative glomerulonephritis
17	Seyrek [19] 1995	Mesangioproliferative glomerulonephritis
18	Sar [20] 2007	Amyloidosis
19	Index case	Diffuse proliferative glomerulonephritis

Glomerular hyperfiltration could play an important role in the development of FSGS and heavy proteinuria in patients with ADPKD. In a histological study of the kidneys of 12 ADPKD patients by Montoyo *et al*. [4], interstitial fibrosis and tubular atrophy were found to be the main determinants of the development of chronic renal failure in ADPKD. In a study of 18 cases, Zeier *et al*. [5] reported interstitial fibrosis and arteriolar sclerosis as being the most important lesions in kidneys of ADPKD patients, whereas FSGS was observed in less than 5% of the glomeruli.

The index case was a diagnosed case of ADPKD who presented with nephrotic-range proteinuria with active urinary sediment. Open kidney biopsy was consistent with DPGN. This association of ADPKD and DPGN has not been described in the literature so far. Proteinuria is usually less than 3 g/24 hours in more than 75% of patients with DPGN, however it may reach the nephrotic range in 20% of hospitalized patients. Open renal biopsy in our case not only aided our prognostications, but also helped us in reaching a definitive diagnosis. Biopsy report enabled us to obviate the use of corticosteroids in our patient, which is the usual modality of empirical therapy in patients with nephrotic syndrome.

To the best of our knowledge, our patient was the first ADPKD patient with nephrotic syndrome caused by post-streptococcal DPGN. It might have been a chance association, as both DPGN and ADPKD are common in the general population, but nephrologists should be aware of this potential association. Our case again illustrates the importance of open renal biopsy in patients with polycystic kidney disease with nephrotic-range proteinuria, as the treatment for various histopathological subtypes leading to nephrotic syndrome is different and in this modern era we should practice evidence-based medicine. It also illustrates a way of avoiding empirical therapy.

## Conclusion

Clinicians are reluctant to perform renal biopsy in patients of ADPKD as it entails an open renal biopsy which requires the involvement of a surgeon. However, our case reinforces the need for renal biopsy in patients with polycystic kidney disease presenting with nephrotic-range proteinuria to exclude any coexisting glomerular disease, and to reach an accurate diagnosis. It also illustrates the importance of open renal biopsy in patients with ADPKD and nephrotic-range proteinuria in order to plan appropriate treatment. Treatment for various histopathological subtypes leading to nephrotic syndrome is different. Corticosteroids are beneficial in some conditions whereas they may not be of any use in other cases. A lesson to be learnt from our case is that an open renal biopsy should be carried out in all patients with ADPKD presenting with nephrotic syndrome to make an accurate diagnosis. Reaching a firm diagnosis based on histopathology and immunofluorescence studies will help the physician to give appropriate treatment in the form of corticosteroids and/or cytotoxic agents, if indicated, and to avoid empirical therapy with these potentially toxic agents and to avoid their possible adverse effects.

## Consent

Written informed consent was obtained from the patient for publication of this case report and any accompanying images. A copy of the written consent is available for review by the Editor-in-Chief of this journal.

## Competing interests

The authors declare that they have no competing interests.

## Authors' contributions

SD was the nephrologist in charge of this patient and was primarily involved in patient management and preparation of the manuscript. RS was the surgeon who performed the open renal biopsy. HM was the histopathologist who reported the kidney biopsy specimen. RK was the radiologist involved in the management of this patient. RWM was the immunopathologist who reported the immunofluorescence of the kidney biopsy specimen. VK assisted in patient management and was involved in manuscript preparation. AS was involved in the manuscript preparation and editing of the text. All authors read and approved the final manuscript.
